# The Health of India

**Published:** 1893-02-11

**Authors:** 


### THE HEALTH OF INDIA.

In the *Calcutta Review* attention is drawn to the recently issued report of the Sanitary Commissioners for Bengal in 1891, as in several respects eminently unsatisfactory. The system of birth registration seems to be curiously, perhaps inevitably, defective. The birth-rate for 141 municipal towns is given at 21·46 per 1,000, with the comment that this is manifestly absurd and impossible, since the true birth-rate cannot be less than 45. Can such a system of registration serve any purpose whatever? The general health of the province is stated to have been decidedly worse in all districts, six towns being alone excepted. The deaths from small-pox were over 16,000, a great increase upon the previous year. This is attributed to defective organisation as regards vaccination. The mortality from fever is also much greater, and this is plainly stated to be due to malarial poison arising from obstructed drainage, polluted water supply, and neglected conservancy arrangements. The terrible mortality from cholera (229,575, or 3·26 per 1,000) is attributed in part to the scanty rainfall in the latter part of the year, but much more to the great movement of the people which took place during February in consequence of the Ardhodoya Jog. This festival depends on the combination of three astronomical conditions which coincide only once in 27 or 28 years, and make the sanctity of the day ten million times as great as that of a sun eclipse. Literally then the stars in their courses fought against the doctors of Bengal.

